# Focusing on persistent hypoxemia during VV-ECMO secondary to traumatic tricuspid valve injury: a case report

**DOI:** 10.3389/fmed.2026.1877495

**Published:** 2026-08-27

**Authors:** Yunpeng Zhao, Jing Liu, Kun Chen

**Affiliations:** Department of Critical Care Medicine, Affiliated Jinhua Hospital Zhejiang University School of Medicine, Jinhua, China

**Keywords:** acute respiratory distress syndrome, infective endocarditis, trauma, tricuspid chordae tendinae rupture, veno-venous extracorporeal membrane oxygenation

## Abstract

**Background:**

Veno-venous extracorporeal membrane oxygenation (VV-ECMO) is a life-saving salvage therapy for treating severe respiratory failure. We present a case of trauma-related hypoxemia with severe tricuspid chordae tendinae rupture managed with VV-ECMO.

**Case presentation:**

We report the successful use of VV-ECMO in a 29-year-old patient with severe acute respiratory distress syndrome (ARDS). Refractory hypoxemia persisted despite maximal conventional therapy during the course of treatment at the outside hospital. Under VV-ECMO support, the patient’s oxygen saturation improved, allowing for the down-titration of ventilator parameters. However, when an attempt was made to reduce the ECMO settings, the patient’s oxygen saturation decreased. Transesophageal echocardiography (TEE) revealed a traumatic rupture of the tricuspid chordae tendineae, which was identified as the underlying etiology of the patient’s severe ARDS. Following surgical repair and subsequent improvement in oxygenation, the patient was successfully decannulated from VV-ECMO. Post-decannulation, the patient developed a high fever, later confirmed as *Burkholderia cepacia* infective endocarditis, which responded well to targeted antimicrobial therapy.

**Conclusion:**

VV-ECMO should be considered a viable supportive therapy for patients with trauma-induced ARDS. However, if acute hypoxemia persists despite conventional interventions during VV-ECMO support, immediate differential diagnosis is warranted. Notably, bedside echocardiography serves as a critical tool for identifying underlying cardiac etiologies of refractory hypoxemia.

## Background

Traumatic tricuspid valve rupture is a rare but life-threatening emergency. If left untreated, massive regurgitation can rapidly precipitate acute respiratory distress syndrome (ARDS) and systemic complications. Veno-venous extracorporeal membrane oxygenation (VV-ECMO) - represents a crucial rescue therapy when conventional treatments fail (1, 2). However, severe tricuspid regurgitation significantly increases the risk of ECMO recirculation, thereby compromising its oxygenation efficiency. The profound hypoxemia observed in this patient could not be attributed solely to pulmonary lesions. In this report, bedside echocardiography successfully identified the underlying cardiac cause of the hypoxemia. Subsequent surgical intervention ultimately enabled the successful weaning of the patient from VV-ECMO, providing valuable clinical insights for the management of similar cases (3).

## Case presentation

A 29-year-old male sustained significant chest and abdominal trauma following a traffic accident and was admitted to a referring hospital on January 15, 2025. He presented with acute hypoxemic respiratory failure (PaO_2_/FiO_2_: 55 mmHg), and subsequently underwent emergency partial splenectomy and mechanical ventilation. The mechanical ventilation was configured in pressure-controlled ventilation-assist/control (PCV-A/C) mode, with a tidal volume of 6 mL/kg predicted body weight, a respiratory rate of 15 breaths/min, FiO_2_ of 100%, PEEP of 15 cmH_2_O, and a plateau pressure of 30 cmH_2_O. The patient could not be placed in the prone position due to severe thoracic and abdominal trauma. In this patient, the decreasing oxygenation index (PaO_2_/FiO_2_) and acute respiratory failure were strong indicators of ARDS and the need for veno-venous extracorporeal membrane oxygenation (VV-ECMO) initiation. However, despite ongoing treatment, it was determined that the patient’s hypoxemia could not be solely attributed to ARDS. Regrettably, echocardiography was not performed at the referring hospital. The second day after the spleen operation, due to refractory hypoxemia, the patient was urgently transferred to our hospital for VV-ECMO. Upon transfer to our hospital for evaluation, the patient had an Injury Severity Score (ISS) of 30 and a Glasgow Coma Scale (GCS) score of E2VTM5. Vital signs revealed a temperature of 36.2°C, a heart rate of 97 beats/min, a blood pressure of 117/64 mmHg, and a respiratory rate of 20 breaths/min. His oxygen saturation was 86% under 100% mechanical ventilation oxygen.

**FIGURE 1:**
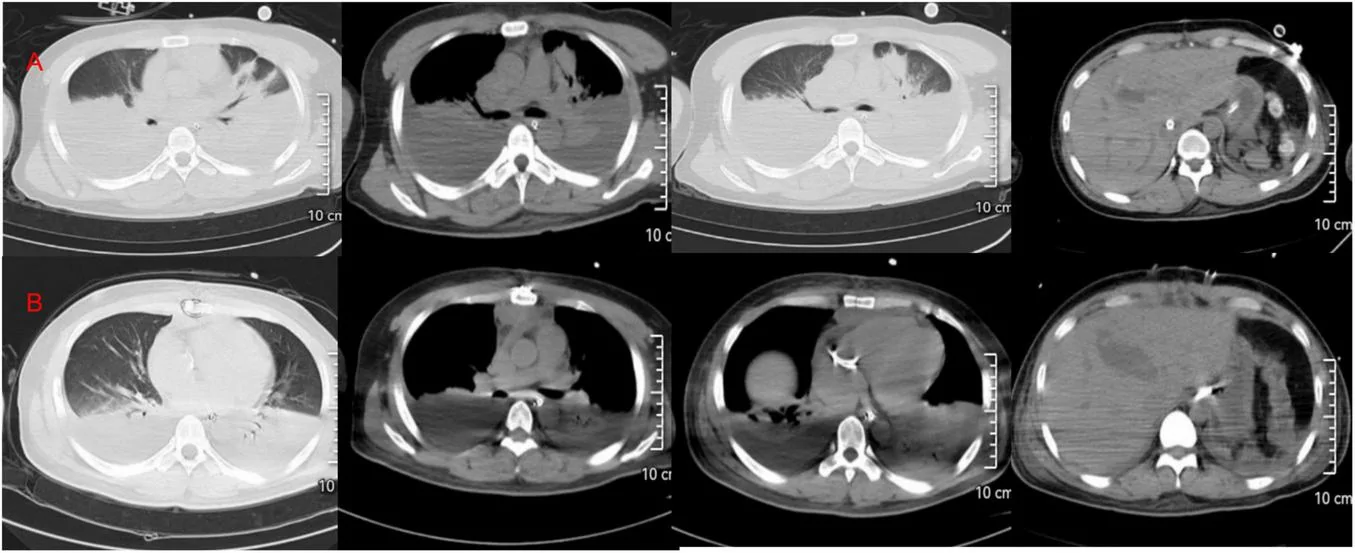
Computed tomography (CT) images of the chest. **(A)** Chest CT performed on the day of transfer to our hospital revealed bilateral patchy high-density shadows and partial bronchial involvement, accompanied by massive bilateral pleural effusions. A small amount of ascites was noted. **(B)** Chest CT performed on postoperative day 3 demonstrated improvement in pulmonary infection and atelectasis, as well as a decrease in bilateral pleural effusions.

**FIGURE 2:**
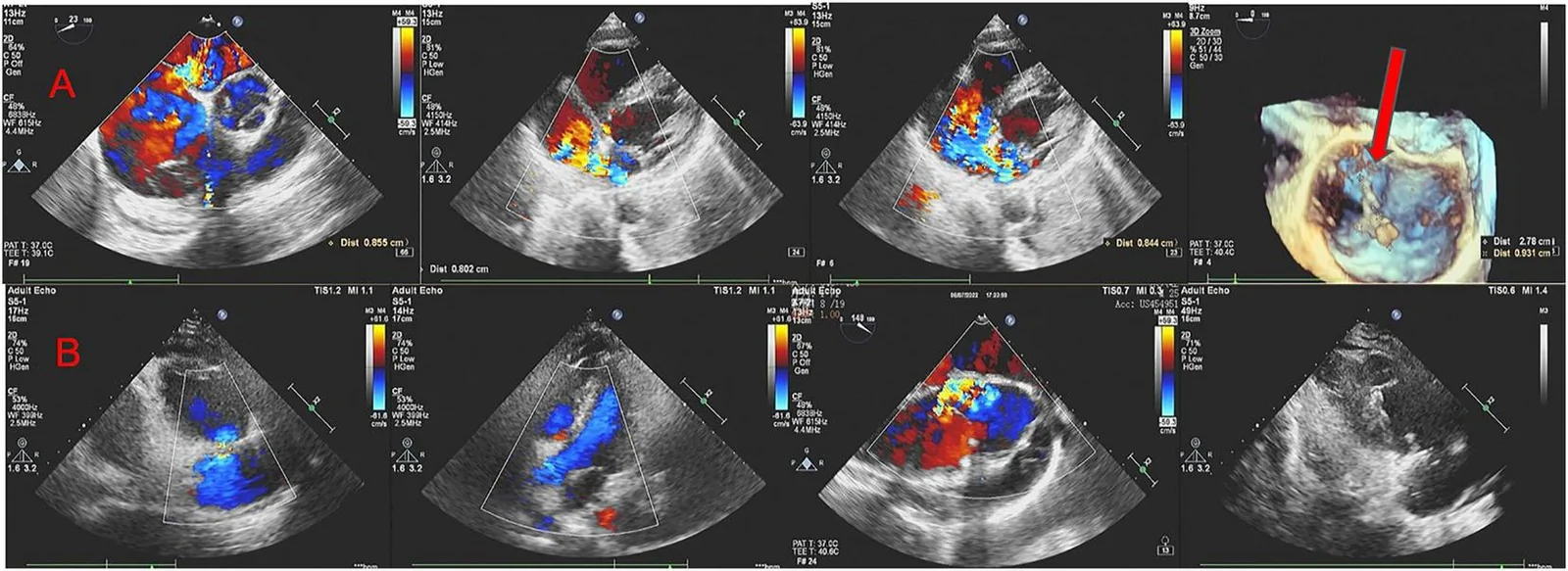
**(A)** Transesophageal echocardiography (TEE) revealed rupture of the tricuspid chordae tendineae with marked tricuspid regurgitation preoperatively (red arrow). **(B)** Three-dimensional TEE (3D-TEE) demonstrated complete resolution of the interatrial shunt and mild tricuspid regurgitation postoperatively.

**FIGURE 3:**
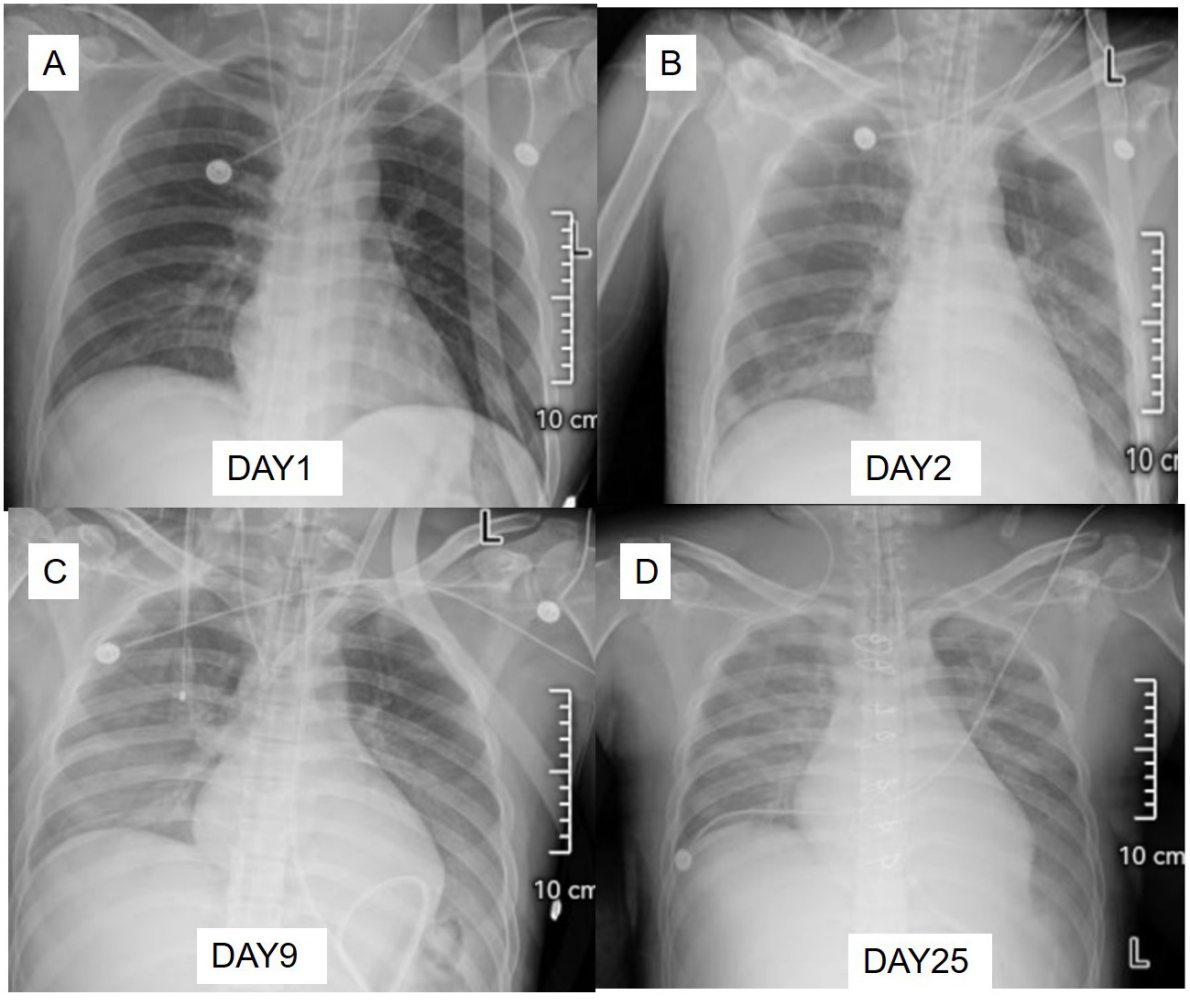
Serial chest radiographs. **(A)** Pre-VV-ECMO (day 1) showing diffuse bilateral pulmonary infiltrates, indicating severe lung involvement. **(B)** Day 2 revealing persistent bilateral infiltrates with worsening consolidation. **(C)** Day 9 demonstrating partial resolution of consolidation and reduction in pulmonary infiltrates. **(D)** Day 25, post-VV-ECMO weaning, showing significant improvement in lung fields with resolved consolidation.

Following ultrasound evaluation of vascular access feasibility and exclusion of contraindications at the puncture sites, VV-ECMO via the right internal jugular (drainage, 17 Fr) and right femoral veins (return, 21 Fr) was initiated on the second day. The initial rotational rate was set at 3000 rpm, blood flow rate of 2.76 L/min, sweep gas flow rate of 5 L/min, and FiO_2_ of 100%. During VV-ECMO support, continuous intravenous heparin was administered for anticoagulation, targeting an Activated Clotting Time (ACT) of 160–200 s and an activated partial thromboplastin time (APTT) of 60–80 s. Following successful VV-ECMO initiation, the oxygen concentration of the ventilator was gradually reduced to 60%, with 10 cmH_2_O PEEP and 25 cmH_2_O plateau pressure. A chest computed tomography (CT) scan revealed pleural effusion and pneumonia on the second day (Figure 1A). Laboratory tests, including body temperature, inflammatory markers, and blood cultures, showed no abnormalities. Following the reduction in blood and oxygen flow from the VV-ECMO, the patient experienced severe hypoxemia immediately. Further investigation revealed an increase in troponin I levels, indicative of myocardial injury. Three-dimensional transesophageal echocardiography (3D-TEE) identified severe tricuspid regurgitation, right atrial enlargement, and bidirectional shunt across an atrial septal defect (Figure 2A). Transesophageal echocardiography (TEE) revealed rupture of the anterior papillary muscle of the tricuspid valve with prolapse of the anterior leaflet. Color Doppler flow imaging (CDFI) demonstrated severe tricuspid regurgitation (grade 4+). Echocardiographic measurements indicated right ventricular dimensions of 60 × 25 mm, right atrial dimensions of 58 × 48 mm, and a main pulmonary artery diameter of 20 mm. Electrocardiography (ECG) revealed ST-segment elevation in the anterior leads, accompanied by elevated troponin levels. Consequently, emergency coronary angiography was performed to rule out coronary artery disease. The procedure confirmed no significant coronary artery stenosis, and the left ventricle exhibited normal contraction with a left ventricular pressure of 120/13 mmHg (mean: 52 mmHg). The cardiac surgeon concluded that the persistent hypoxemia was due to abnormal intracardiac hemodynamics, necessitating surgical intervention. After confirming no contraindications for surgery, tricuspid valve repair and atrial septal defect closure were performed surgery were performed on the 11th day. Intraoperative findings revealed that rupture of the chordae tendineae attached to the anterior papillary muscle resulted in prolapse of the entire anterior leaflet of the tricuspid valve. Blood stasis was observed on the epicardial surface, and a 2-cm atrial septal defect was identified. The anterior leaflet was reconstructed using artificial chordae and stabilized with a rigid annuloplasty ring. Concurrently, the atrial septal defect was repaired. Postoperative TEE demonstrated only trivial tricuspid regurgitation and a significant reduction in the interatrial shunt. After surgical treatment, the patient’s pulmonary oxygenation progressively improved. Postoperative echocardiography demonstrated a reduction in the atrial shunt and the appearance of multiple short and indistinct echodensities echoes on the right atrial surface of the anterior leaflet of the tricuspid valve, accompanied by mild tricuspid regurgitation (Figure 2B). As the parameters of VV-ECMO gradually declined, the patient’s oxygen saturation remained stable. On the second day after cardiac surgery, VV-ECMO blood flow rate decreased to 1.5 L/min, while ventilator FiO_2_ decreased to 40%, with 6 cmH_2_O PEEP and 22 cmH_2_O plateau pressure, maintaining at ≥95% SpO_2_. Chest X-ray results demonstrated an improvement in the lung condition, and the VV-ECMO weaning trial was successfully passed. Consequently, the patient’s VV-ECMO was successfully removed on the 12th day. Postoperative surveillance CT revealed an improvement in pulmonary infection and a reduction in pleural effusion (Figures 1B, 3).

**FIGURE 4:**
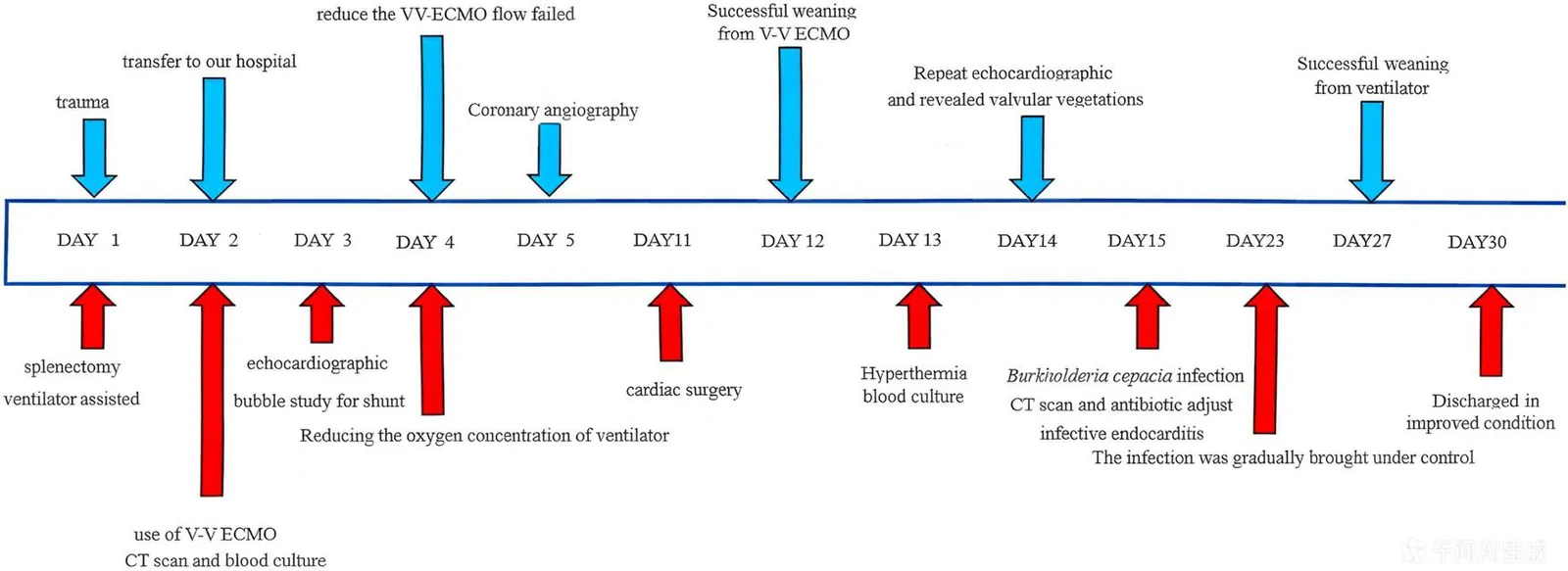
Patient’s treatment course.

Following the VV-ECMO decannulation, the patient’s oxygen saturation was maintained, although the inflammatory markers progressively worsened, accompanied by elevated white blood cell count (28.17 × 10^9/L), neutrophil count (22.08 × 10^9/L), C-reactive protein (61.74 mg/L), and procalcitonin (2.37 ng/mL) (Table 1). Concurrently, the patient was promptly referred for further diagnostic workup, including a blood culture test. On the 15th day, the blood cultures identified *Burkholderia cepacia* as the causative organism. TEE imaging revealed an irregular hyperechoic mass measuring approximately 18 × 7.2 mm on the surface of the anterior tricuspid leaflet, associated with the tricuspid valve (Figure 2B). TEE revealed multiple short, villous, and fluffy echogenicities on the right atrial surface of the anterior tricuspid leaflet. These findings were highly suggestive of vegetations, indicating a diagnosis of infective endocarditis (IE) (4, 5). The patient was started on empirical antimicrobial therapy comprising intravenous meropenem (2 g every 8 h), ceftazidime (2 g every 8 h), and sulfamethoxazole/trimethoprim (15 mg/kg/day), targeting both community-acquired and healthcare-associated pathogens. Following 72 hours of treatment, there was significant clinical improvement marked by sustained afebrility (temperature < 37.8 °C), reduction of inflammatory biomarkers (C-reactive protein decreased from 105.69 mg/L to 35.59 mg/L), and negative serial blood cultures. Hemodynamic stability was maintained without vasopressor support, with persistent normalization of lactate levels (<2 mmol/L). Repeat chest radiography demonstrated progressive resolution of bilateral alveolar infiltrates and improved pulmonary aeration, consistent with radiographic response to antimicrobial intervention. Following the completion of a 6-week targeted antimicrobial course, the patient was successfully weaned from mechanical ventilation and transferred to a rehabilitation facility. Figure 4 illustrates the clinical timeline and major management interventions.

**TABLE 1:** Dynamic changes of oxygenation parameters in laboratory, ventilator and VV-ECMO before start-up, before operation, after operation and after extracorporeal membrane oxygenation offline.

Clinical parameters	Pre-ECMO	ECMO day 1	ECMO day 2	POD -1	POD 0	POD 1	Remove ventilator
Ventilatory parameters							
FiO_2_	100%	100%	60%	40%	45%	55%	50%
Respiratory rate	15	20	17	15	16	15	17
PEEP (cm H_2_O)	15	10	10	12	10	8	–
Tidal volume (mL)	391	306	412	422	395	426	–
Gas analysis							
PH	7.298	7.557	7.445	7.407	7.251	7.415	7.516
PCO_2_ (mmHg)	55.8	23.2	29.1	36.4	26.9	27.4	35.4
PO_2_ (mmHg)	59.6	119.8	77.9	83.1	116.1	119.2	79.3
BE	−7.2	−2.1	−5.8	−2.3	−10.6	−0.5	5.1
HCO_3_-(mmol/L)	14.2	20.2	18.6	22.4	15.2	24.1	28
Lac (mmol/L)	8.1	3.65	2.15	1.57	2.41	3.25	1.34
ECMO record							
ECMO ration rate (revolutions/min)	–	3000	3000	3000	2000	–	–
ECMO flux (L/min)	–	2.76	3.5	3.7	2.7	–	–
ECMO FiO_2_	–	100%	100%	100%	100%	–	–
ECMO sweep gas flow rate (L/min)	–	5	3	3	3	–	–
Laboratory inspection							
WBC (10^9/L)	20.55	18.44	18.41	15.35	17.26	27.69	25.11
CRP (mg/L)	198.56	114.03	55.42	105.69	95.41	93.34	35.59
PCT (ng/ml)	2.92	2.03	1.37	1.11	0.96	7.77	2.84
APTT(s)	49.6	67.3	65.2	63.5	62.5	59.4	38.9

FiO_2_, fraction of inspired oxygen; PCO_2_, partial pressure of carbon dioxide; PO_2_, partial pressure of oxygen; Lac, lactate; BE, base excess; WBC, white blood cell; CRP, C-reactive protein; PCT, procalcitonin; APTT, activated partial thromboplastin time. POD -1: One day prior to tricuspid valve repair and atrial septal defect closure. POD 0: On the day of tricuspid valve repair and atrial septal defect closure operation. POD 1: One day after the tricuspid valve repair and atrial septal defect closure operation.

## Discussion

Despite optimized mechanical ventilation, the patient experienced inadequate gas exchange secondary to severe pulmonary dysfunction, necessitating the initiation of VV-ECMO as rescue therapy. VV-ECMO is an extracorporeal life support modality that diverts systemic venous blood through a membrane oxygenator to facilitate gas exchange independent of intrinsic pulmonary function. This mechanism augments systemic oxygen delivery and is particularly effective when conventional positive-pressure ventilation, even with adjunctive prone positioning, fails to achieve target arterial oxygenation. VV-ECMO is primarily indicated for acute hypoxemic respiratory failure with preserved cardiac function, as it mitigates ventilator-induced lung injury and enables lung-protective ventilation strategies. In the present case, the patient met the Berlin criteria for ARDS and exhibited refractory hypoxemia despite maximized ventilatory support, thereby warranting VV-ECMO as a critical component of multimodal salvage therapy (6, 7).

In resource-limited centers, early TEE evaluation of trauma patients is often hindered by hemodynamic instability, high transport risks, and a lack of specialized bedside imaging personnel. However, early echocardiography is crucial when severe hypoxemia develops post-trauma. Beyond pulmonary etiologies such as airway obstruction and lung injury, bedside transthoracic echocardiography can rapidly identify or rule out underlying cardiogenic factors.

The reduced efficacy of VV-ECMO can be attributed to multiple factors. After excluding mechanical failures of the oxygenator and circuit, a systematic evaluation should be performed. First, increased patient oxygen consumption–driven by fever, chills, or seizures–can compromise VV-ECMO efficacy. Second, an excessively high native cardiac output may result in an insufficient VV-ECMO-to-cardiac output ratio, leading to recirculation, where oxygenated ECMO blood is immediately re-aspirated into the circuit. Third, inadequate drainage can significantly reduce the performance of the membrane oxygenator. If these factors are ruled out, cannula malposition or recirculation must be considered. In such cases, chest X-ray should be utilized to re-evaluate the position of the drainage or reinfusion cannulas, alongside an assessment of the difference between pre-oxygenator saturation (SpO_2_-pre) and the patient’s mixed venous oxygen saturation (SvO_2_). Additionally, echocardiography is recommended to evaluate structural cardiac changes.

Transesophageal echocardiography revealed rupture of the tricuspid chordae tendineae accompanied by severe tricuspid regurgitation (TR) and a traumatic atrial septal defect (ASD). During VV-ECMO support, oxygenated blood was reinfused into the right atrium via the internal jugular vein and subsequently entered the right ventricle. However, severe TR caused a significant portion of this oxygenated blood to regurgitate back into the right atrium during ventricular systole, leading to right atrial dilatation (58 × 50 mm) and elevated right atrial pressure (>15 mmHg). Concurrently, deoxygenated blood traversed the pulmonary circulation and entered the left atrium. Due to the concurrent severe pulmonary infection and pleural effusion, pulmonary gas exchange was severely impaired, resulting in inadequate oxygenation. This poorly oxygenated blood then shunted back into the right atrium through the ASD. This complex hemodynamic derangement significantly compromised the overall oxygenation efficiency of the VV-ECMO circuit. Surgical repair of the tricuspid valve and atrial septum was successfully performed. Following surgical correction of these anatomical abnormalities, VV-ECMO oxygenation efficiency was markedly restored. This improvement was evidenced by an increase in the PaO_2_/FiO_2_ ratio from 155 mmHg to 265 mmHg within 24 h. Surgical repair of the atrial septum and tricuspid valve was successfully performed. On postoperative day 1, the patient successfully passed the VV-ECMO weaning trial, maintaining stable oxygen saturation without the need to adjust ventilator parameters. These clinical improvements met the criteria for successful VV-ECMO decannulation (8).

Two blood cultures collected 24 h apart grew *Burkholderia cepacia*. Echocardiography demonstrated tricuspid valve vegetations, fulfilling two major modified Duke criteria. Furthermore, predisposing factors and fever (>38 °C) satisfied two minor criteria. Based on these findings, a definitive diagnosis of IE was established (4, 9). However, the diagnostic yield of IE is often suboptimal due to the low sensitivity of blood cultures, which can lead to delays in diagnosis. This is particularly relevant in our case, as blood cultures were negative upon admission. TEE of the anterior tricuspid valve leaflet revealed echodense changes, suggesting bacterial colonization of the damaged valve. Although blood cultures were negative upon admission, they subsequently yielded *Burkholderia cepacia* following tricuspid valvuloplasty (10). This organism is a peroxidase-positive, aerobic, non-lactose-fermenting, Gram-negative bacillus ubiquitous in soil and water environments, predominantly affecting immunocompromised individuals (11, 12). IE caused by *Burkholderia cepacia* is exceedingly rare; as of 2024, only 20 cases have been reported in the literature (13, 14). For instance, Almubarak et al. described a case of *Burkholderia cepacia* IE involving the aortic valve, which was successfully managed with initial empirical antibiotic therapy followed by urgent surgical aortic valve replacement, ultimately resolving the patient’s aortic insufficiency (13). In this case, severe systemic trauma from a traffic accident predisposed the patient to *Burkholderia cepacia* infection. The trauma-induced structural damage to the tricuspid valve, specifically the ruptured chordae tendineae, provided an ideal nidus for bacterial colonization and dissemination. Following the diagnosis, a combined antibiotic regimen effectively suppressed bacterial growth, leading to the gradual resolution of fever and a decline in inflammatory markers. Historically, *Burkholderia cepacia* IE is most frequently identified in individuals who use intravenous drugs. Additionally, a literature review evaluating ECMO as a preoperative bridge for IE yielded a cohort of 22 patients. Pooled analysis demonstrated an overall in-hospital survival rate of 81.8% (18/22) with ECMO bridging. Furthermore, patients who underwent surgical treatment exhibited a significantly higher survival rate compared to those receiving conservative management (94.4% vs. 25%) (14).

Although traumatic tricuspid regurgitation (TTR) is a rare entity, accounting for only 0.02% of traumatic injuries, it may be underdiagnosed due to its non-specific and often clinically silent presentation. In recent years, the reported incidence of TTR appears to be increasing, likely attributable to advancements in echocardiographic techniques and the rising global prevalence of traffic accidents. Due to its immediate retrosternal position, the right ventricle is particularly vulnerable to deceleration forces during blunt chest trauma. The most frequently reported injuries involve the rupture of chordae tendineae, followed by anterior papillary muscle rupture and tears of the anterior tricuspid leaflet. Jacob et al. reported a case of tricuspid papillary muscle rupture complicated by cardiogenic shock following a motor vehicle accident. In that patient, severe TR caused an acute elevation in right atrial pressure, which subsequently reduced left ventricular preload and cardiac output, manifesting as severe hypotension. The patient was successfully treated with veno-arterial (VA) ECMO as a bridge to surgical repair. Our case shares similar traumatic pathophysiology but differs significantly due to the concomitant traumatic ASD. In our patient, the regurgitant blood could shunt across the ASD into the left atrium, preventing a sustained increase in right atrial pressure. Consequently, hemodynamic stability was preserved, whereas respiratory failure predominated. While ECMO serves as a crucial bridge for acute cardiopulmonary failure, VV-ECMO is primarily indicated for isolated respiratory failure. In cases complicated by severe circulatory collapse, transitioning to VA or VVA configurations is warranted (15).

## Conclusion

This case report demonstrates the successful application of VV-ECMO in managing traumatic tricuspid regurgitation complicated by severe ARDS. The rapid improvement of ARDS via VV-ECMO, combined with the use of transthoracic echocardiography and TEE to identify the underlying causes of ECMO inefficiency, holds significant clinical value for patients with post-traumatic hypoxemia. This case highlights the potential of this integrated approach in similar clinical scenarios. Further research is warranted to validate its broader applicability and efficacy.

## Data Availability

The datasets presented in this study can be found in online repositories. The names of the repository/repositories and accession number(s) can be found in the article/supplementary material.
